# A New Approach to Carbon Nanotube Filament Nanostructuring for Additive Manufacturing

**DOI:** 10.3390/polym16101423

**Published:** 2024-05-17

**Authors:** Fedor Doronin, Mikhail Savel’ev, Georgy Rytikov, Andrey Evdokimov, Victor Nazarov

**Affiliations:** Faculty of Printing Industry, Moscow Polytechnic University, 107023 Moscow, Russia110505n@gmail.com (V.N.)

**Keywords:** filament, additive manufacturing, carbon nanotubes, nanostructured mathematical modeling, polymer substrate, surface modification

## Abstract

A new technique of additive prototyping filament volumetric nanostructuring based on the high-speed mechanical mixing of acrylonitrile-butadiene-styrene (ABS) copolymer granules and single-walled carbon nanotube (CNT) powder (without prior dispersion in solvents) is considered. The morphological spectra of scanning electron microscopy (SEM) images of nanostructured filament slice surfaces were obtained and characterized with the original mathematical simulation. The relations of structural changes in the “ingredient-matrix” polymer system with dielectric and mechanical properties of the ABS-based filaments were established. The supplementation of 1.5 mass.% of CNT powder to the ABS filament composition leads to the tensile strength increasing from 36 ± 2 to 42 ± 2 MPa. It is shown that the greater the average biharmonic amplitude and the morphological spectrum localization radius of the slice surfaces’ SEM images, the lower the electrical resistance of the corresponding nanostructured filaments. The possibility of carbon nanotube-modified filament functional layers forming using the extrusion additive prototyping technique (FFF) on the surface of plasma-chemically modified PET substrates (for the creation of load cell elements) is experimentally demonstrated.

## 1. Introduction

The development of new technologies for the formation of microchannel systems and sensors on solid and flexible polymer and polymer composite substrates is an important area of modern materials science [1]. There is a need to develop wearable microelectronics [2] (primarily for medical purposes) which form the prerequisites for creating sensors and transducers based on flexible polymers [3,4,5] using 3D prototyping [6] or/and high-performance printing technologies [7]. Fundamental and applied scientific achievements in the field of additive (printing) technologies [8] provide an opportunity for the world’s leading manufacturers to produce high-performance photonics and lighting products: solar cells, LEDs, photosensitive arrays, etc. [9,10].

Among the set of additive technologies with a well-known range of advantages, the most promising one is 3D prototyping using the extrusion FFF technique [11]. This is a consequence of the low cost of the polymers (and some composites [12]) and the substrate (filament) and the absence of material loss during product molding.

Polyethylene-terephthalate-glycol (PETG) [13], polylactide (PLA) [14], and the acrylonitrile-butadiene-styrene copolymer (ABS) [15] seem to be useable for the creation of semiconductors and conductive polymer-based elements with extrusion 3D printing. However, the low interlayer adhesion of the filament and the peeling of the formed prototypes from the heated 3D printer platform can lead to critical product defects [16]. Moreover, typical filaments do not always and fully satisfy the technological requirements of the original components when used in repair processes (for example, due to the differences between the physics-chemical characteristics of metals and polymers). Thus, it is necessary to regulate both the technological parameters of the extrusion additive prototyping [17] and the properties of surface-textured polymer substrates for the high-quality and accurate manufacturing of 3D-printed products.

The development of polymer composites with the improved properties set is an urgent scientific and technical task [18]. It is known that the composite filaments have the enhanced (compared to unfilled materials) mechanical [19,20], electrical [21,22] and other properties [23,24,25]. The relevance of physics-chemical, functional and operational properties’ direct regulation with volumetric [26] and the surface modification [27,28] is due to the possibility of the significant changes in the polymers’ structure.

There is a demand for polymer composites with enhanced thermal and electrical conductivity for robotic devices, unmanned transport systems, and high-tech medical and printing equipment [29]. Carbon nanotubes (CNTs) have recently been actively used as fillers [30,31] in creating conductive polymer-based compositions. The stability of CNT-filled filament and corresponding product properties is provided by the homogeneity of the filler spatial distribution over the polymer matrix volume [32]. Uneven distribution leads to void formation (misprinting) and 3D-printed layer displacement (smearing). This is a critical drawback that prevents the implementation of additive prototyping in flexible electronic component manufacturing [33].

Free surface energy changes (caused by the transformations of the chemical composition, structure, and/or microtexture of the polymer materials) can provide both strengthening and an increase in electrical conductivity. The efficiency of polymer waste destruction processes (for example, with bacteria and/or fungi [34]) is largely determined by the hydrophilicity and uniformity of water condensate distribution over the product’s surface. Knowledge of the functional surface properties of polymers is of great importance for their use in a wide range of technologies, including paints, coatings, and biocompatible materials for transplantology, and for solving certain problems of microfluidics [35]. The surface characteristics of the materials are taken into account when creating nanotechnologies intended for the development of specialized devices on a flexible polymer base [36]. 

The most universal technique of polymer material free surface energy control is plasma-chemical treatment [37,38]. The use of plasma-chemical treatment (especially in the presence of the oxygen) leads to a decrease in the water contact angle by several times for polymers such as polycarbonate, polyvinylidene fluoride, polystyrene, and polyimide [39]. However, the achieved effect of wetting angle reduction is not always stable over time [40]. This instability is apparently associated with the peculiarities of the plasma-chemical process. Its primary actions are the breaking of chemical bonds and the formation of free radicals in the polymer structure. Over time, these radicals undergo chemical (including oxidative) and recombination transformations, leading to the cross-linking and the destruction of the polymer, the formation of unsaturated bonds, etc. This should be taken into account when forming 3D-printed and other structures on the polymer surface.

We propose a new technique for the volumetric nanostructuring of additive prototyping filaments with carbon nanotubes. It is based on the high-speed mechanical mixing of ABS granules and CNT powder without the prior long-term dispersion of carbon nanotubes in solvents. We also show that plasma-chemical treatment allows the reliable coating of CNT-modified ABS filament functional layer on the surface of a polyethylene terephthalate (PET) substrate.

## 2. Materials and Methods

**Figure 1:**
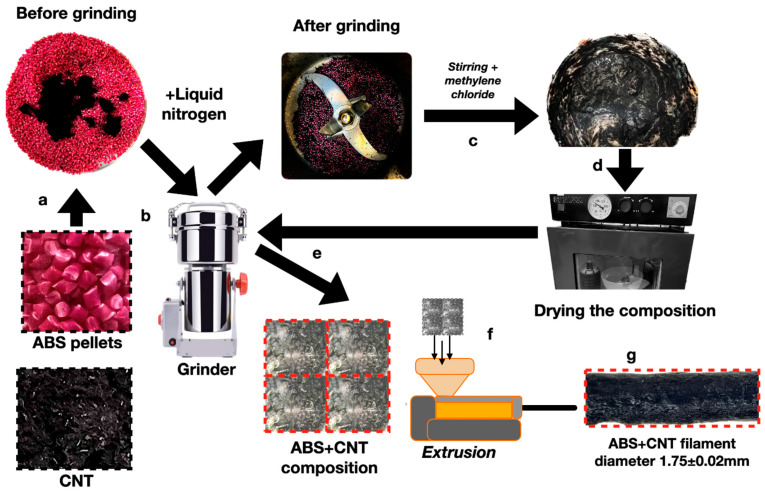
The scheme of the CNT-volume-modified ABS filament thread fabrication. The letters a–g indicate the technological operations described in the text. a—mixing composition (ABS pellets and CNT) before grinding; b—grinding the composition; c—stirring the composition with methylene chloride; d—dry composition after stirring; e—grinding dry composition; f—extrusion of filament; g—ABS+CNT filament.

We carried out the volumetric nanostructuring of a filament with various concentrations (0.5, 1.5, 3.0, and 5.0 mass.%) of Tuball OCSiAl carbon nanotubes by mixing a melt of acrylonitrile-butadiene-styrene copolymer (3D Systems, Rock Hill, SC, USA) with CNT powder in a single-screw extruder (Filastruder, Atlanta, GA, USA) at a temperature of 240 °C, according to the procedure of Vasilyev [41]. The composition mixtures of ABS and CNTs (weighing ~100 g) were prepared according to the scheme shown in Figure 1 by means of the mechanical grinding and mixing of ABS granules and CNT powder (Figure 1a) in a grinder (Figure 1b) at a blade rotation speed of 38,000 rpm, with the addition of 100 mL of liquid nitrogen to prevent the premature melting of the polymer material due to the heating of the grinder’s metal walls (for 15 s). Next, 50 mL of methylene chloride (chemically pure) was added to the crushed ABS CNT composition (Figure 1c), and stirring was carried out (for 5 min) in the overhead stirrer Heidolph Hei TORQUE Precision200 (Heidolph Instruments GmbH&CO. KG, Schwabach, Germany) until a homogeneous substance was obtained. This significantly reduced the duration of the preparation of ABS-CNT compositions in comparison to the method of [42] due to the absence of the preliminary stage of the ultrasonic dispersion of CNTs in various liquid media. The drying of the ABS-CNT composition (necessary to remove the methylene chloride) was carried out for 6 h at a temperature of 100 °C in a ZEAMiL HORIZONT SPT-2000 vacuum oven (Poland) (Figure 1d). Milling in the grinder (Figure 1e) was carried out to enable the extrusion formation (Figure 1f) of the CNT-filled ABS filament thread (Figure 1g).

**Figure 2:**
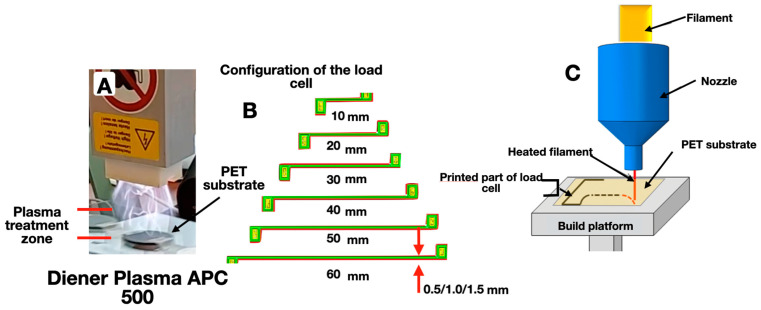
Installation for directed physicochemical design through the plasma-chemical treatment of the surface of polymer substrates used in extrusion 3D prototyping (**A**); configuration of strain gauge elements (**B**) and schematic diagram [43] of extrusion FFF 3D printing (**C**).

An empirical study of the volumetrically modified filaments’ morphological characteristics was carried out using the scanning electron microscope JSM-7500 FA (JEOL, Akishima-shi, Japan), operated in the secondary electron detection mode at an accelerating voltage of 10 kV, and using an Oxford X- max 80 detector with a SATW-window at accelerating voltages of 10 and 20 kV at an electron current of ~1 nA. When optimizing for the spectral characteristics of silicon, the sensitivity of the device ranged from 0.2 (for oxygen) to 1.0 (for carbon) atomic percent. Thus, the average depth of analysis calculated in the Win Casino v2.48 program using the Monte Carlo method was (for 10 kV) ~0.4 µm. The average measurement error did not exceed 2%. A HoldPeak HP890CN digital multimeter (HoldPeak, Zhuhai, China) was used to measure the electrical resistance of the experimental samples. The mechanical properties of the volume-modified filament were studied using the ZwickRoell BZ1.0/TH1S Universal Tensile Testing Machine (178579/2007) (Zwick GmbH & Co. KG, Ulm, Germany). The modification of PET substrates with a thickness of 20, 140, and 300 microns was carried out in the specialized air-plasma system Diener Plasma APC 500 (Diener electronic GmbH&Co. KG, Ebhausen, Germany). The surface energy (γ_s_) calculation (polar γ_s_^P^ and dispersive γ_s_^D^ components, mJ/m^2^) was carried out using the Owens–Wendt–Rabel–Kaelble (OWRK) method based on the determination of wetting edge angles (Θ°) for distilled water (Θ°water) and ethylene glycol (Θ°eg) using the installation KSVCAM 101 (KSV Instruments, Helsinki, Finland). The extrusion FFF 3D printing of microfluidic strain gauges, with electroconducting line lengths from 10 to 60 mm and thicknesses of 0.5, 1.0, and 1.5 mm (Figure 2), was carried out on an Anycubic Mega S 3D printer (Shenzhen, China) with ABS filaments filled with carbon nanotubes, at a temperature of 260 °C and a nozzle diameter of 400 microns.

**Figure 3:**
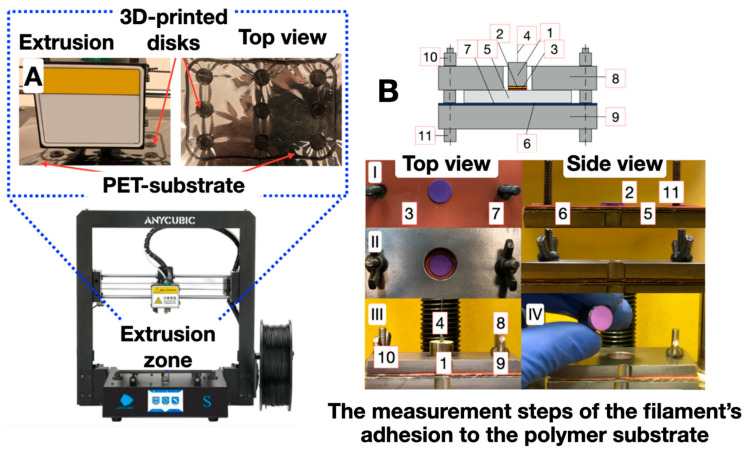
Scheme of the test object’s adhesion determining (**A**) a “filament–polymer substrate” pair using a specialized bursting machine (**B**). Measurement steps (I–IV) of the filament’s adhesion to the polymer substrate: 1—metal cylinder with an adhesive layer; 2—test object; 3—PET substrate; 4—Kevlar twisted thread; 5—tested sample; 6—double-sided adhesive tape; 7—rubber gasket; 8—upper steel plate with a hole for the metal cylinder; 9—lower steel plate; 10—nut; 11—bolt [16].

To determine the peel strength σ, test objects in the form of disks with an area of 1 cm^2^ were made from PLA filament on the surface of a PET substrate (Figure 3). Next, a metal cylinder with the butt area of 1 cm^2^ was fixed to the test object using cyanoacrylate glue “Moment” (“Henkel”), which was attached to the upper clamp of an Instron 3382 tensile testing machine by means of a flexible rod. The values of the tensile strength were recorded using the StretchTest program [16].

## 3. Results and Discussion

### 3.1. Creation of CNT-Nanostructured Filaments for Additive Manufacturing

**Figure 4:**
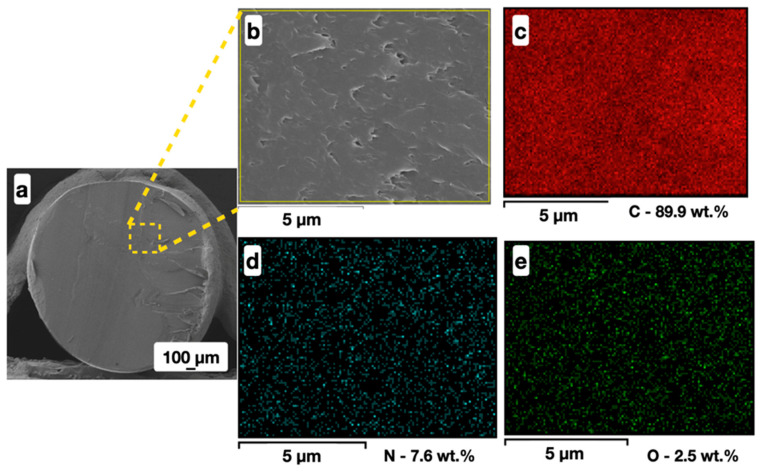
SEM images (**a**,**b**) and planar distribution of elements (C—(**c**); N—(**d**); and O—(**e**)) over the surface of the CNT-volume-modified ABS filament thread cross-section.

A cross-section of an unfilled ABS filament has a homogeneous surface structure (Figure 4a,b), the elemental composition of which corresponds to an acrylonitrile-butadiene-styrene copolymer (Figure 4c–e). The presence of oxygen (~2.5 wt.%) in the unfilled ABS filament is due to the long-term storage of the granules in natural conditions and to thermal oxidation under the extrusion process [44].

The dispersion of CNTs in various solvents is one of the main preparatory operations for the manufacture of filaments filled with carbon nanotubes (the “wet method” [45,46,47]). It is necessary to obtain a suspension with a high concentration of individual (non-agglomerate) nanotubes. The filament (obtained with the “wet method”) is characterized by a high degree of diameter instability [48]. This worsens the interlayer adhesion when preparing products using 3D printing. The multistage and long-term process of obtaining structurally homogeneous filaments filled with carbon nanotubes (implying the use of expensive extrusion rheometers [49]) is described by Podsiadły [42] and Verma [50]. The ultrasonic dispersion of CNTs in acetone was carried out for 20 min to destroy the agglomerates. The ABS granules were added to the suspension. The composition was mixed in a magnetic stirrer for 5 h. The ABS/CNT composition was dried in vivo at room temperature for 24 h to evaporate the solvent. The granulate from the ABS-CNT composition was additionally dried at a temperature of 100 °C for 60 min for the final evaporation of the acetone.

The specified drying time was still insufficient to remove the acetone from the composition [50]. The remaining solvent caused the formation of pores in the filament. It eventually led to critical defects in the composite filament. The manufacturing process had to be repeated four times according to the “extrusion–granulation–extrusion” scheme in order to obtain a structurally homogeneous ABS-CNT thread with a diameter of 1.7 mm. Thus, it took more than 30 h to obtain an ABS-CNT filament with a high degree of uniformity in the concentration of components [50]. Other “wet methods” for obtaining composite materials were developed [51,52,53,54]. The preparation of polystyrene-based composites with the pre-dispersion of CNTs (0.0025 wt.%) in 300 mL of CHCl_3_ in a 70 W ultrasonic homogenizer for 30 min, followed by drying the suspension in a desiccator at 400 °C until the complete evaporation of the solvent, was described in [51].

A multistage technology for the production of polyvinylidene fluoride-based PTFE/CNT composites (with a number of thread extrusion technological limitations) was proposed by Almazrouei [52]. It consists of the pretreatment of composite powders with ultrasound in deionized water and ethanol (under various technological conditions) and the production of composite sheets (a 500 kg plate measuring 6 × 6 inches, at a temperature of 170 °C for 10 min) using a pressing machine.

The technique of obtaining polybutylene terephthalate-based PBT/CNT composites for electrically conductive surface structure additive prototyping was described by Gnanasekaran [53]. CNT dispersions were pre-prepared in 100 mL of isopropanol and treated with ultrasound for 2 h in an ice bath to prevent heating. PBT was added to the mixture and it was additionally treated with ultrasound for 60 min. Isopropanol was evaporated in a water bath at a temperature of 90 °C. The resulting PBT/CNT composition was dried at room temperature for 24 h. 

The technology for producing polylactide-based PLA/CNT composites was proposed by Xu [54]. It consists in dissolving PLA granules in dichloromethane, mixing CNT powder using a magnetic stirrer, and drying the resulting composition in a fume hood.

We have developed a technology for the volumetric nanostructuring of acrylonitrile butadiene styrene with carbon nanotube powder. It consists of the high-speed mechanical mixing of ABS granules and CNT powder without the prior long-term dispersion of carbon nanotubes in solvents.

**Figure 5:**
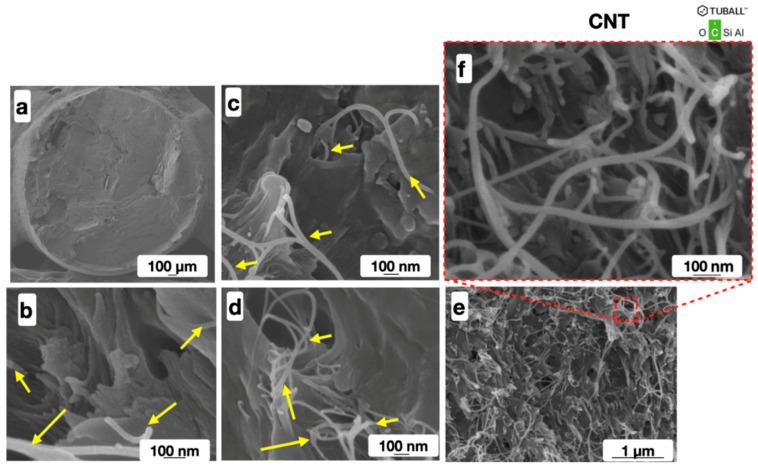
SEM images of the carbon nanotube-filled ABS filament (**a**) with 0.5 mass.% (**b**); 1.5 mass.% (**c**); 3.0 mass.% (**d**); and 5.0 mass.% (**e**,**f**) of CNTs. Yellow arrows show the carbon nanotubes.

**Figure 6:**
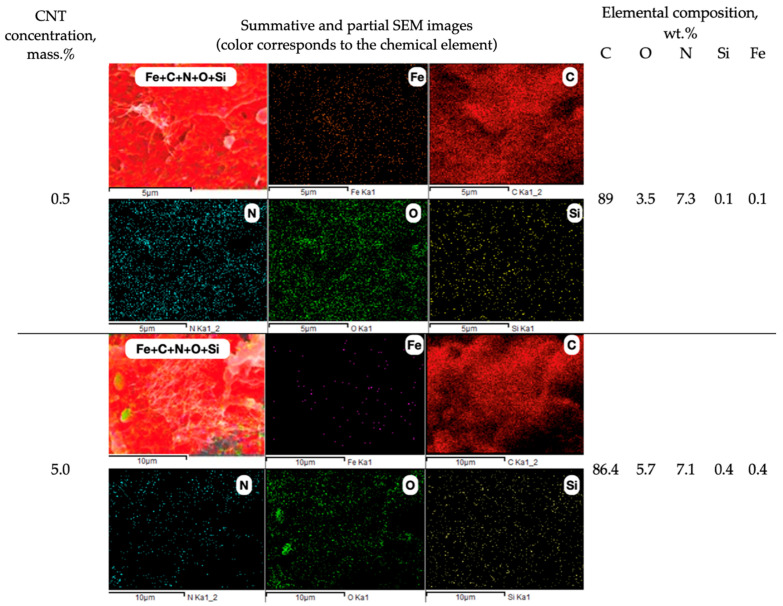
Chemical element (C, N, O, Fe, and Si) plane distribution over the CNT-modified ABS filament thread cross-section surface.

As a result, a macroscopically homogeneous ABS/CNT filament for additive prototyping (Figure 5a) was obtained. Its micro- and nanostructure were studied using the SEM (Figure 5b–f) and EDS (Figure 6) techniques.

The EDS images (Figure 6) of chemical element distributions in the cross-sectional plane of the filament thread make it possible to observe the changes in the degree of homogeneity for carbon (C), nitrogen (N), oxygen (O), iron (Fe), and silicon (Si), with an increase in modifier content in the polymer matrix. The presence of Fe and Si in the composition is explained by the fact that the CNT powder contains up to 15% of metallic and other impurities. The Fe and Si content in the ABS-based composite increases proportionally from 0.1 to 0.4 wt.% with an increase in CNTs from 0.5 to 5 mass.%; and the amount of C decreases from 89.9 (Figure 4c) to 86.4 wt.%.

**Figure 7:**
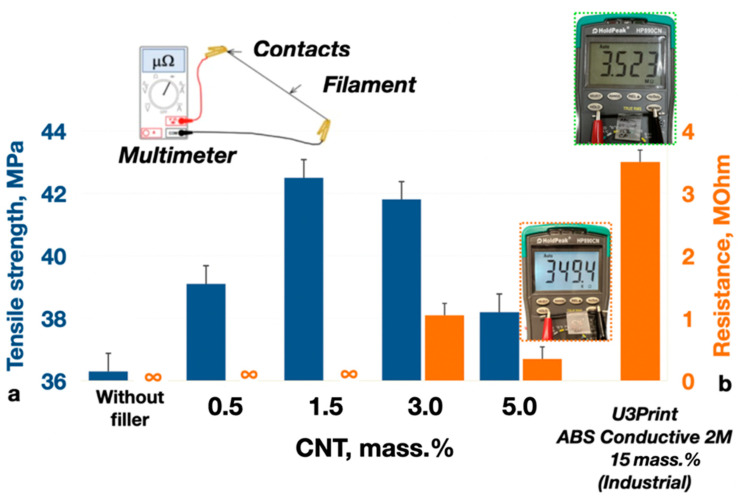
The effect of the carbon nanotube content onto the tensile strength (**a**) and electrical resistance (**b**) of the CNT-modified ABS filament thread (diameter—1.75 ± 0.02 mm; length—4 cm).

The changes in the carbon distribution over the polymer matrix (Figure 6) (caused by the volumetric modification of ABS with the carbon nanotubes) significantly affect the electrical and mechanical properties of the composite material (Figure 7b).

The introduction of 1.5 mass.% of CNT powder to the ABS filament composition leads to the tensile strength increasing from 36 ± 2 to 42 ± 2 MPa. The volumetric modification of the ABS filament with 5 mass.% of CNTs decreases the electric resistance from 3.4 (commercially available U3Print ABS Conductive 2M filament, containing 15 mass.% of CNTs) to 0.3 MOhm (~10 times). The original technique [55,56], based on the digital representation of polymeric material surface SEM images in a two-dimensional trigonometric Fourier series form, was used for the analytical characterization of the CNT-nanostructured ABS filaments:

(1) $$I\left(x,y\right)≅\sum_{k,l}I_{kl}=\sum_{k,l}\left\{{\left(\begin{array}{c} \begin{array}{c} a_{kl}\\ b_{kl}\\ c_{kl} \end{array}\\ d_{kl} \end{array}\right)}^{T}\cdot \left(\begin{array}{c} \cos\left(2\pi \cdot k\cdot x/L_{x}\right)\\ \sin\left(2\pi \cdot k\cdot x/L_{x}\right) \end{array}\right)\times \left(\begin{array}{c} \cos\left(2\pi \cdot l\cdot y/L_{y}\right)\\ \sin\left(2\pi \cdot l\cdot y/L_{y}\right) \end{array}\right)\right\}$$

where *k*, *l* are the biharmonic order indices; *a_kl_*, *b_kl_*, *c_kl_*, and *d_kl_* are the partial amplitudes; and ((2*π*·*k*·*x*)/*L*_x_) are the partial phases of the biharmonic components.

Each mathematical expression *I_kl_* can be associated with a regular pattern characterized by two spatial periods (*L_x_* and *L_y_*). The additive set of such patterns forms the material structure 3D model. So the main textural–morphological matrix characteristic of the experimental samples’ surfaces is the morphological spectrum in which components (amplitudes) can be approximately calculated with the equation:

(2) $$A_{kl}≅\sqrt{a_{kl}^{2}+b_{kl}^{2}+c_{kl}^{2}+d_{kl}^{2}}$$

**Figure 8:**
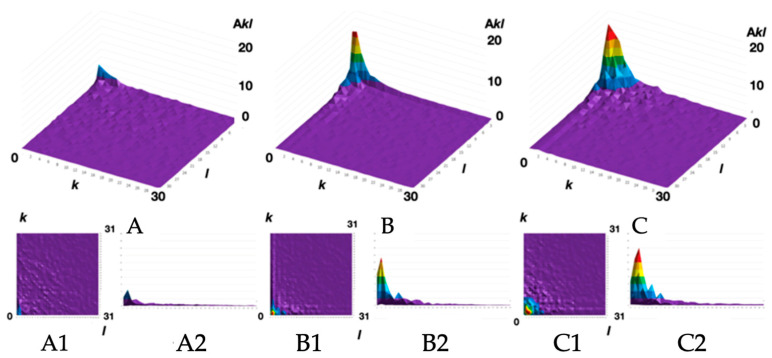
The morphological spectra A_kl_ (**A**–**C**), their projections onto the spatial plane of the lattice wave vectors k and l (**A1**,**B1**,**C1**), and their profilograms A_kl_ (k) (**A2**,**B2**,**C2**) for the ABS filaments: unfilled (**A**) and nanostructured with 3 mass.% (**B**) and 5 mass.% (**C**) of CNTs.

The morphological spectra (obtained for the SEM images of the experimental samples’ surfaces) are shown in Figure 8.

**Figure 9:**
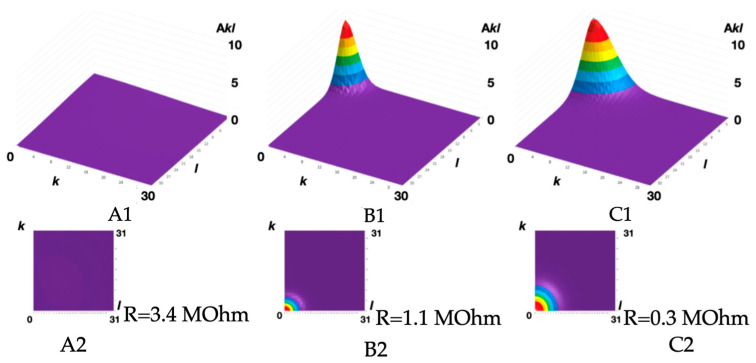
The morphological spectrum Gaussian models (**A1**,**B1**,**C1**) and their projections onto the spatial plane of the lattice wave vectors k and l (**A2**,**B2**,**C2**) with the electrical resistance values R for the obtained ABS filaments: unfilled (**A**) and nanostructured with 3 mass.% (**B**) and 5 mass.% (**C**) of CNTs.

It can be seen (Figure 8) that an increase in the content of carbon nanotubes in the filament leads to an increase in the average amplitude and an expansion of the morphological spectrum localization area. The morphological spectra were approximated by a two-dimensional Gaussian function for a quantitative description of these changes in the polymeric materials’ surface structure. The Gaussian function maximum value determines the average amplitude, and the characteristic size of its flat section in 1/e level is the localization area radius (Figure 9).

So we have established (Figure 9) that the larger the morphological spectrum localization area radius and the average biharmonic amplitude, the lower the electrical resistance of the ABS filament thread which is volumetrically modified with CNTs.

### 3.2. CNT-Nanostructured Filaments for Additive Manufacturing Elements of Load Cells on Modified PET Substrates

**Figure 10:**
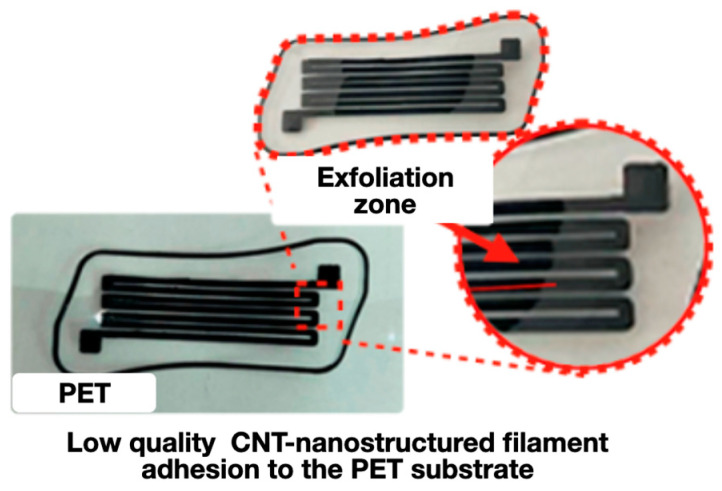
Printing defects caused by filament exfoliation due to insufficient adhesion to the initial (unmodified) PET substrate.

It is obvious that the initial polymer substrates are of little use for the manufacture of strain gauges using extrusion additive technologies, since the adhesion of the filament to their surface is insufficient [38]. The adhesive and functional properties of 3D-printed sensors are also affected by the configuration of the corresponding printed elements (see Figure 10).

There are printing defects on the polymer substrates’ surface (Figure 10) caused by the exfoliation of the filament layers due to low adhesion to PET as a result of the non-optimal design and configuration of printed strain gauges.

Since the amount of adhesion and the uniformity of the application of filament layers to polymer substrates directly depend on the structure (morphology) and chemical composition of their surface, as well as on the modification parameters (duration), we monitored these parameters with high accuracy before and after modification [38].

**Figure 11:**
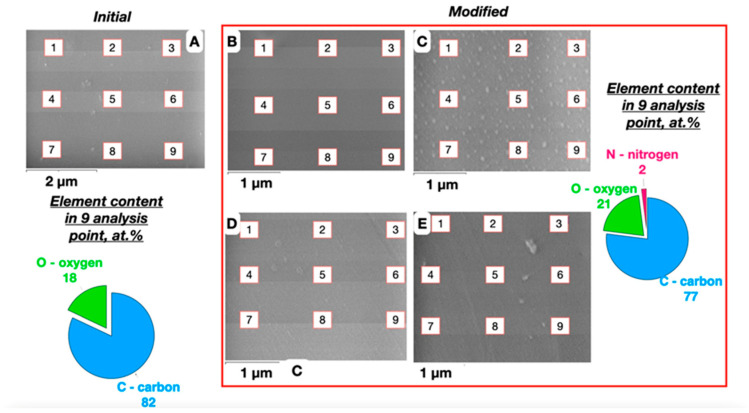
SEM and EDS analysis (C—carbon; N—nitrogen; and O—oxygen) of the surface of the original (**A**) and plasma-chemically treated PET substrates for 15 (**B**); 30 (**C**); 45 (**D**); and 60 (**E**) seconds [38].

It can be seen (Figure 11) that the change in the chemical composition of the near-surface and surface layers of the PET substrates during plasma-chemical treatment is associated with the oxidizing activity of the plasma, which correlates with the EDS data (Figure 11B–D)—the increase in the duration of the modification contributes to an increase in oxygen content from 18 to 21 at.%. and, accordingly, oxygen-containing functional groups (the presence of nitrogen in the amount of 2 at.% is due to its presence in the air).

**Figure 12:**
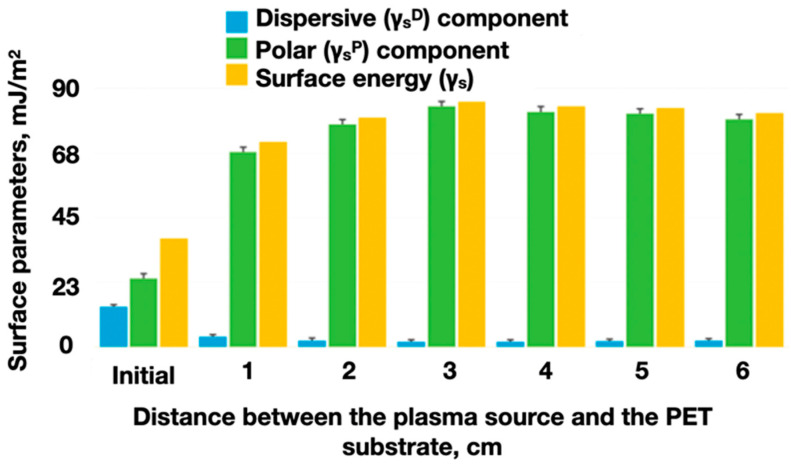
Values of surface energy (γ_s_) and its polar (γ_s_^P^) and dispersive (γ_s_^D^) components depending on the distance between the plasma source and the PET substrate with a treatment duration of 60 s; 0—initial (unmodified) PET; 1–6—distance between the plasma source and the PET surface (cm).

The plasma-chemical treatment of PET substrates (Figure 12) is most effective at a distance of 3–6 cm from the plasma source, because at smaller distances, a significant thermal effect on the polymers occurs, and at larger distances, the impact of the plasma arc on the polymer surface is insufficient.

**Figure 13:**
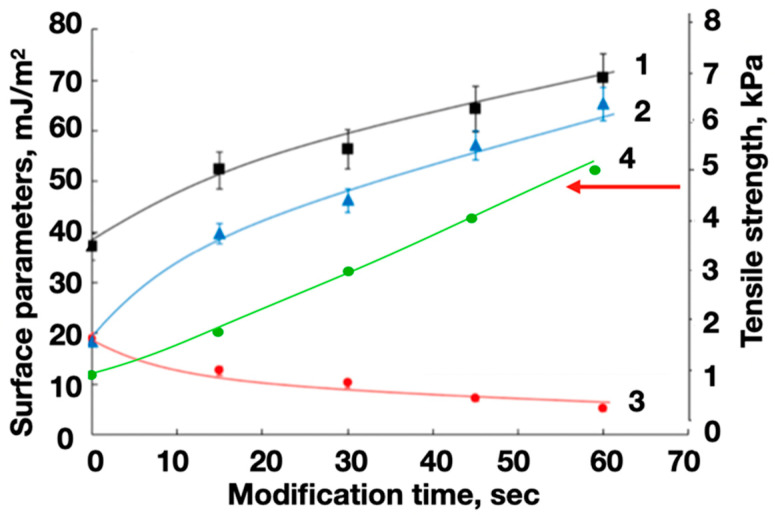
The dependences of the surface energy γ_s_ (1) and its polar γ_s_^P^ (2) and dispersive γ_s_^D^ (3) components for PET substrates on the duration of plasma-chemical treatment and the tensile strength (4) for the separation of the ABS filament test objects from the PET surface.

A significant (Figure 13) change in the surface energy of the PET substrates (on average 2 times) contributed to a proportional increase in the adhesion of the functional layers of the ABS filament.

**Figure 14:**
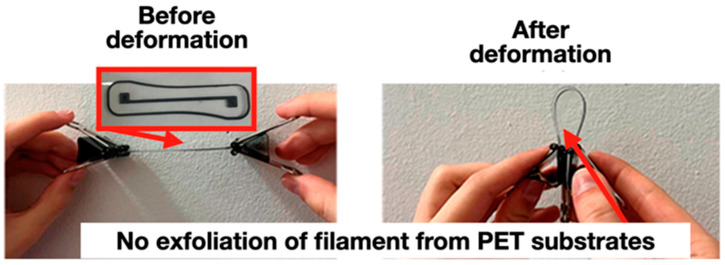
Strain gauge element on the surface of a modified PET substrate with reliable fixation of ABS filament layers (no filament peeling; for comparison, see Figure 10).

It was revealed that the strength of the adhesive interaction of electrically conductive filaments based on ABS and PET substrates with a functional oxygen-containing layer increased by more than 4.5 times compared to the original (unmodified) substrates (Figure 14). This will ensure the reliable formation of bending sensor elements (gauge elements) using extrusion additive prototyping.

## 4. Conclusions

We propose a new approach to the bulk modification of ABS filaments with CNTs to eliminate the preliminary stage of individual ingredient dispersion when manufacturing electrically conductive and semiconductor components of flexible electronics and planar photonics using additive prototyping technologies. 

The morphological transformations of the ABS copolymer matrix caused by CNT nanotexturing contributed to a decrease in the electrical resistance and an increase in the mechanical strength of the created composite materials. 

The control of the technological parameters of the plasma-chemical treatment of PET substrates contributed to the high-quality application of 3D-printed strain gauge elements (bending sensors) on their surfaces using extrusion additive prototyping. 

We revealed that the strength of the adhesive interaction of electrically conductive filaments based on ABS and PET substrates with a functional oxygen-containing layer increased by more than 4.5 times compared to the original (unmodified) substrates.

This provides an opportunity for the development and improvement of industrial additive technologies (3D printing) when manufacturing different high-tech devices (electrochemical sensors, microhydrodynamic contactors, glucose meters, and radio-absorbing materials) on a flexible polymer basis.

## Data Availability

Data are contained within the article.
